# Age constraints for the Trachilos footprints from Crete

**DOI:** 10.1038/s41598-021-98618-0

**Published:** 2021-10-11

**Authors:** Uwe Kirscher, Haytham El Atfy, Andreas Gärtner, Edoardo Dallanave, Philipp Munz, Grzegorz Niedźwiedzki, Athanassios Athanassiou, Charalampos Fassoulas, Ulf Linnemann, Mandy Hofmann, Matthew Bennett, Per Erik Ahlberg, Madelaine Böhme

**Affiliations:** 1 Department of Geosciences, Eberhard Karls University, Tübingen, 72076 Tübingen, Germany; 2 Senckenberg Centre for Human Evolution and Palaeoenvironment, Tübingen, Germany; 3 Department of Geology, Faculty of Science, Mansoura University, Mansoura, 35516 Egypt; 4 Senckenberg Naturhistorische Sammlungen Dresden, Museum Für Mineralogie Und Geologie, Sektion Geochronologie, Königsbrücker Landstraße 159, 01109 Dresden, Germany; 5 Faculty of Geosciences, University of Bremen, Klagenfurterstr. 2-4, 28359 Bremen, Germany; 6 Department of Organismal Biology, Uppsala University, Norbyvägen 18A, 752 36 Uppsala, Sweden; 7 Hellenic Ministry of Culture and Sports, Ephorate of Palaeoanthropology-Speleology, Ardittou 34B, 11636 Athens, Greece; 8 University of Crete, Natural History Museum, 71409 Iraklion, Greece; 9 Department of Life and Environmental Sciences, Bournemouth University, Bournmouth, UK

**Keywords:** Palaeomagnetism, Geochemistry, Palaeontology

## Abstract

We present an updated time frame for the 30 m thick late Miocene sedimentary Trachilos section from the island of Crete that contains the potentially oldest hominin footprints. The section is characterized by normal magnetic polarity. New and published foraminifera biostratigraphy results suggest an age of the section within the Mediterranean biozone MMi13d, younger than ~ 6.4 Ma. Calcareous nannoplankton data from sediments exposed near Trachilos and belonging to the same sub-basin indicate deposition during calcareous nannofossil biozone CN9bB, between 6.023 and 6.727 Ma. By integrating the magneto- and biostratigraphic data we correlate the Trachilos section with normal polarity Chron C3An.1n, between 6.272 and 6.023 Ma. Using cyclostratigraphic data based on magnetic susceptibility, we constrain the Trachilos footprints age at ~ 6.05 Ma, roughly 0.35 Ma older than previously thought. Some uncertainty remains related to an inaccessible interval of ~ 8 m section and the possibility that the normal polarity might represent the slightly older Chron C3An.2n. Sediment accumulation rate and biostratigraphic arguments, however, stand against these points and favor a deposition during Chron C3An.1n.

## Introduction

**Figure 1:**
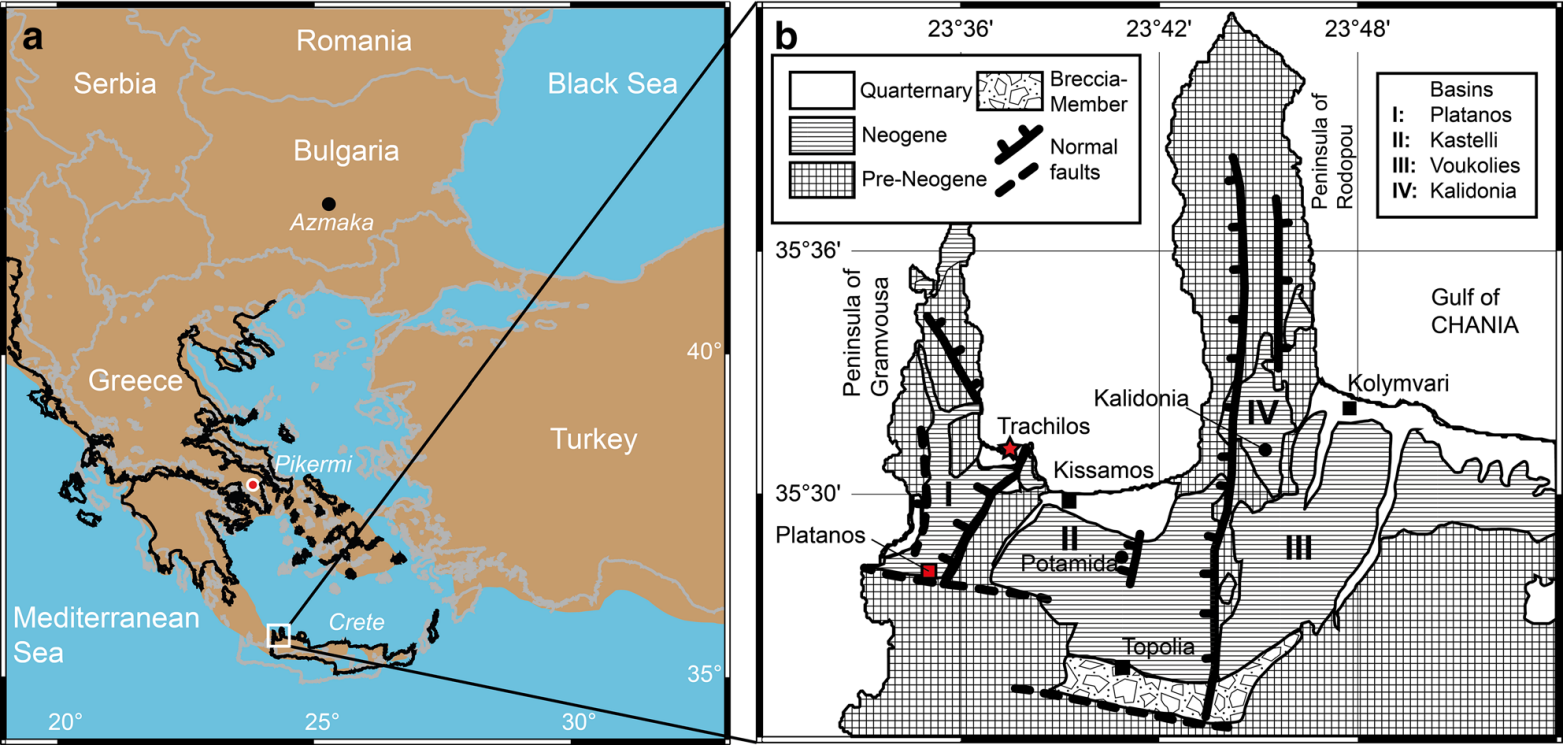
(**a**) Map of the study location on Crete (white square) together with the Aegean Sea. Black coastline, brown land and blue sea surface is based on reconstructions of the area for 6.0 Ma^11^. Circles show the position of the hominid sites of Pyrgos Vassilissis (close to Pikermi) and Azmaka^1^. (**b**) Local geological map of the study area in north-western Crete^8^ showing the main deposition basins and the Trachilos section (red star). Maps were created using GMT5^12^ (https://www.generic-mapping-tools.org/).

The evolutionary history and dispersal patterns of hominins are matters of debate^1^. One unresolved aspect is the origin and identification of the first representatives of the hominin lineage^2^. Despite numerous publications suggesting an origin in Africa e.g.,^2–5^, there are evidences that the earliest hominins might have evolved in Eurasia^6,7^. Evidence for a Miocene hominin presence in Europe includes both body and trace fossils. Fuss et al.^7^ identified *Graecopithecus freybergi* from Pyrgos Vassilissis near Athens as a probable hominin on the basis of its dentognathic morphology, while Gierliński et al.^6^ presented a morphometric analysis of footprints found on the island of Crete (Trachilos section, Fig. 1, S1). This section belongs to the Platanos Basin and the Vrysses Group of northwestern Crete (Fig. 1b)^8^.

These footprints, which possess a suite of characteristic trace fossil features including expulsion rims, pull-up structures and toe drag marks, indicate the track-maker had a distinctive foot morphology. This morphology includes characters that are currently considered be unique to hominins such as the presence of a forefoot ball, a non-divergent and robust hallux placed alongside digit II on the distal margin of the sole and digits II through IV becoming progressively shorter. These are combined with generic primate traits such as the absence of a longitudinal medial arch, a proportionately shorter sole and a heel that is not bulbous. A straightforward comparative morphological analysis of the character suite suggests that the track-maker was a primitive hominin and a strong case has been made for it being phylogenetically basal to the Laetoli trackmaker, which had a longer, more human-like sole^9^. The morphometric analysis of Gierliński et al.^6^, shows that the Trachilos footprints cluster in the same anatomical space with other hominin footprints and are clearly separated from non-hominin primates.

This interpretation has been controversial, and several counter-interpretations have been made. For example, Meldrum and Sarmiento^10^ suggested that the Trachilos tracks may have been made by a non-hominin primate with an adducted hallux and they illustrated this with reference to a gorilla footprint. We believe that this comparison actually reinforces the interpretation of Gierliński et al.^6^. The illustrated track lacks a ball print, has a hallux print set back from digit II and separated from it by a substantial gap, and the strongly oblique concave posterodistal margin of the sole print reflects the length and finger-like character of digits II through V. None of these features are matched in the Trachilos footprints, which instead resemble the human footprint used in illustration by Meldrum and Sarmiento^10^. So, while we are mindful of the need for caution in the absence of any body fossils, the case that the Trachilos track-maker can be identified provisionally as a primitive, bipedal hominin has not been disproven.

The characteristics of these tracks, together with their geographical location and supposed age, potentially make them highly informative about early hominin evolution^13–15^. However, at present their scientific significance is limited by the poor age control on the site^13^. To address properly the exact importance of these findings and to put them into a global context, especially with respect to Africa, absolute ages are vital.

Gierliński et al.^6^ constrained the youngest possible age by stratigraphic relationship with the overlying conglomerate, which they interpreted as the Hellenikon Group^16^, deposited during the Messinian Salinity Crisis (MSC) between 5.60 and 5.53 Ma^17^. However, neither Frydas and Keupp^8^ nor Zachariasse et al.^18^ identified Hellenikon Group sediments in the Platanos sub-basin, and so the overlying cover in Trachilos is more likely related to cemented Pleistocene beach conglomerates. This leaves planktonic foraminifera as the remaining constraining factor for the age of the Trachilos footprints with a rather large range between 8.5 and 3.5 Ma^6^. With the wrong assumption of the stratigraphic proximity of the footprints below the Hellenikon Group, Gierliński et al.^6^ conclude a likely age close to the onset of the MSC at ca. 5.7 Ma. Without information on the missing time between the Trachilos sediments and the MSC, however, a much older age is also possible. To improve the age resolution and obtain additional information on the sediment provenance, we conducted a magnetostratigraphic study together with foraminifera and detrital zircon analyses.

## Geologic setting and sampling

**Figure 2:**
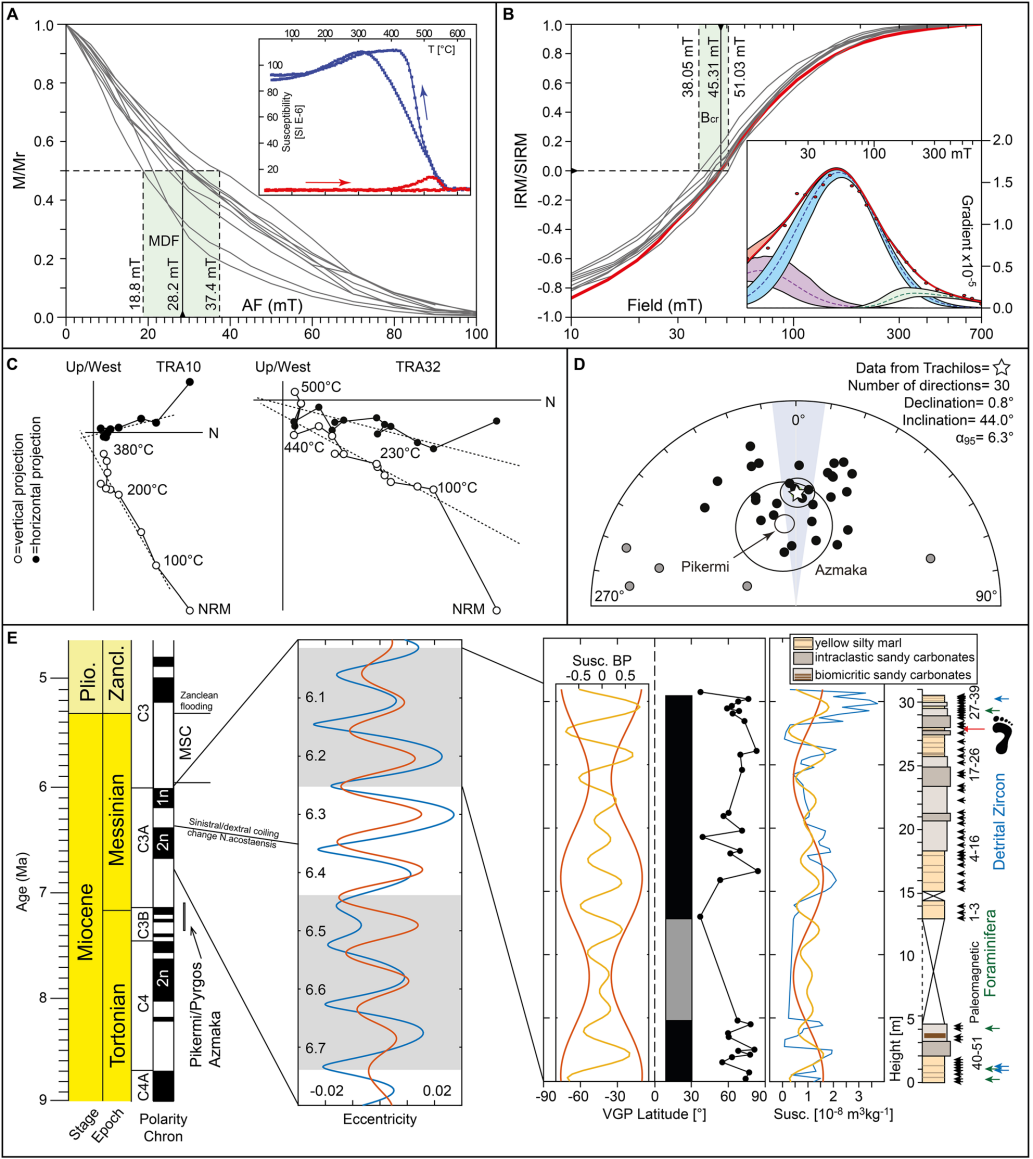
Paleomagnetic and cyclostratigraphic results of the Trachilos section. (**a**) AF demagnetization of ARM (normalized) with indication of the median destructive field (MDF), representative susceptibility vs. temperature curves are shown in the inset; (**b**) backfield IRM acquisition curves normalized for the saturation IRM (SIRM) with indication of the coercivity of remanence (B_cr_); skewed generalized Gaussian-based IRM unmixing of a representative curve (red) (see supplementary information for further detail). (**c**) representative thermal demagnetization results. (**d**) Stereographic projection including results from Pikermi and Azmaka; a_95_ = 95% confidence angle (Fisher, 1953), grey dots are discarded using a 45° virtual geomagnetic pole (VGP) cutoff. (**e**) From left to right: Timescale with stages and magnetic polarity for the 9.0–5.0 Ma interval of the late Miocene-Pliocene^25^. ~ 100 ka bandpass filter for the northern hemisphere insolation curve for the 6.0–6.8 Ma interval^26^. Plot of VGP latitude for the Trachilos samples with according polarity interpretation (black equals normal polarity and grey equals interval with no information), with the bandpass filtered susceptibility record. Susceptibility record (raw data in blue) together with two bandpass filters (red and yellow). Lithological column with positions of paleomagnetic (black), foraminifera (green) and detrital zircon (blue) samples.

The late Tortonian to early Messinian shallow marine sedimentary rocks of the Platanos Basin crop out at Trachilos Beach along 30.5 m of stratigraphic section composed of alternating yellow marls, grey calcarenites and bioclastic limestones^8^. It stratigraphically overlies a deep water facies, which has been correlated to the Kissamou Formation of the Potamida section (Fig. 1^8^). Magnetostratigraphy and cyclostratigraphy constrain the age of the Potamida section at the Tortonian–Messinian boundary^19^. The high quality of the magnetic signal at Potamida is related to the presence of biogenic magnetite^20^. At its top, the Trachilos Beach section contains the footprints described by Gierliński et al.^6^. Based on calcareous nannoplankton^8^, corresponding sediments of the Platanos section ~ 7 km south of Trachilos (Fig. 1b) were correlated with biozone CN9bB between 6.8 and 6.0 Ma^21^, predating the onset of the MSC at 5.971 Ma^22,23^, which led to the total disappearance of foraminifers and (shortly afterwards) nannoplankton from the Mediterranean Basin. To further constrain the age of the Trachilos footprints, we performed a combined paleomagnetic and micropaleontologic study of the section exposed at Trachilos Beach. We extended the ~ 18 m section of Gierliński et al.^6^ to 30.5 m and 57 oriented samples were obtained with a resolution of 20 to 100 cm (Fig. 2). In the lower part of the section there is a sampling gap of ~ 8 m between ~ 5 and 13 m stratigraphic height (Fig. 2). This part was not accessible for sampling because it was covered by sea water. It was however visible that the section is continuous (Fig. S1). Additionally, in four of the 57 paleomagnetic samples, planktonic foraminifera were identified, and three extra samples were taken for detrital zircon analysis: two at stratigraphic level 2 m from a couplet of fine grained yellow silty marl (Trachilos-A) and coarser grained calcarenites (Trachilos-B), and one at 28 m (Trachilos-C) (Fig. 2).

## Results

### Paleo- and rock magnetism

Results from alternating field (AF) demagnetization of anhysteretic remanent magnetizations (ARM) and from stepwise isothermal remanent magnetization (IRM) acquisition indicate the presence of low magnetic coercivity magnetite likely of bacterial origin (Fig. 2c + d).

Reliable paleomagnetic data were obtained from 61% of the 57 samples, yielding exclusively normal polarity directions (Fig. 2a, S2). The natural remanent magnetization (NRM) is weak (less than 0.1 mA/m in ~ 80% of samples, Fig. S3). The combination of unblocking temperature of the characteristic remanent magnetization (ChRM) and rock magnetic results suggest remanence carriers likely represented by biogenic magnetite (Fig. 2). Combining all mean directions of the Trachilos section yields a mean direction of D = 0.8°, I = 44.0° and α_95_ of 6.3° based on 35 samples (Fig. 2b). This result would imply a paleolatitude of only 25.8° (uncertainty = 21.1°–31.1°), which is low compared to the most recent paleogeographic reconstructions for the region (Fig. 1^11^). Comparison with paleomagnetic results from Pikermi and Azmaka^1^, reveals similar declination values, but shows that especially the red sediments of the Pikermi Formation give a paleolatitude estimate of 37.3° (41.4°–33.6°), in agreement with its present-day location (and the reconstruction for the late Miocene). The ChRM directions from Trachilos samples have likely experienced some degree of inclination shallowing, commonly observed in sediments^24^.

### Cyclostratigraphic results

Spectral analysis based on magnetic susceptibility yields a long wavelength peak of ~ 1500 cm (Fig. 2, S4, S5), whose statistical significance remain low due to the presence of only two cycles within the whole section. The fact that the second significant peak of ~ 400 cm (Fig. 2, S2, S3) reveals amplitude modulation using both Fast-Fourier transform (FFT) and multi-taper approaches in agreement with the long wavelength cycle adds confidence that both cycles represent actual signals. This result has to be treated with caution, however, since the data gap between ~ 5 and 13 m stratigraphic height might facilitate the node of the ~ 400 cm amplitude modulation.

### Biostratigraphic results

Foraminiferal analysis shows that there is generally a low diversity trend among planktonic forms, with an exceptionally high abundance of *Orbulina* (Fig. S6) which reaches 80–90% of the total count, characteristic for assemblages just prior the MSC^27,28^. In addition, identification of *Turborotalita multiloba* in our samples (Fig. S6) suggest an age after its First Common Occurrence (FCO) at 6.42 Ma^28^*.* Coiling directions of *Neogloboquadrina acostaensis* reveal the presence of both sinistral and dextral coiled *Neogloboquadrinas* suggesting an age after the coiling change at 6.35 Ma^28^, before which there are almost exclusively sinistral coiled *Neogloboquadrinas*. Coiling direction ratios of *N. acostaensis* show an abundance of sinistral over dextral forms, except for only one sample which totally lacks dextral ones (Fig. S6). These bioevents, especially the abundance of *Orbulina* and the occurrence of *T. multiloba* suggest a deposition during the late MMi13 Mediterranean planktonic foraminiferal zone, between 6.35 Ma and the disappearance of planktonic foraminifera in the Mediterranean at 6.01–5.99 Ma^28^. These results agree with calcareous nannoplankton biostratigraphy of the Platanos section in the south of the Platanos Basin (Fig. 1b)^8^, where Frydas and Keupp^8^ described *Nicklithus amplificus* and *Amaurolithus delicatus*. They suggest that *N. amplificus* can be related to the calcareous nannofossil biozone CN9bB between 6.727 and 6.023 Ma^21,29^. *A. delicatus* has its first occurrence in the Mediterranean at 6.415 Ma^22^, which suggests an age range of the Trachilos sedimentary formations of 6.415–6.023 Ma.

### Detrital zircon geochronology results

Detrital zircon geochronology from all three Trachilos samples yield a distribution with most occurring ages between ~ 500 and 800–900 Ma and some Paleoproterozoic components suggesting a provenance related to the Cadomian, Avalonian and Pan-African orogenies (Fig. S7; zircon ages summarized in^30–33^). Only the coarser grained calcarenite sample (Trachilos B, Fig. S7) yielded an additional Paleozoic peak centered at around 350 Ma and some Mesoproterozoic detritus, typically observed in the northeastern realm of the Mediterranean^30^. Unfortunately, no Neogene zircon grains were identified, which could add maximum age constraints to our age model. We interpret the observed zircon age populations as being dominated by an eolian input from northwestern Africa, e.g., the West African Craton^32^, similar to what has been observed in Pikermi^1^. Only when there is an input of coarser grained material in calcarenites, potentially related to storm events, can a local Tethys or possibly Pelagonian-Cycladic signal be observed^30^.

## Discussion

Although the paleomagnetic signal is stable in the studied sediments (Fig. 2, S2), the quality is not as high as observed in the Potamida clay^19^. A reason for this might be a more favorable environment for magnetotactic bacteria during the deep water phase in Potamida compared to the very shallow environment at Trachilos^20^. The influx of biogenic magnetite related to production of magnetotactic bacteria during climatic variations might also be the reason why it seems possible to detect Milankovitch cycles in Trachilos sediments using magnetic susceptibility. Alternatively, the observed susceptibility variations might be explained by changes in eolian influx of high coercivity/low susceptibility magnetic minerals during cold phases compared to low coercivity/high susceptibility magnetite dominated influx during warmer periods^34^. A connection of the susceptibility signal to eolian influx during intensified desert periods of the Sahara desert is supported by our detrital zircon results, which might in turn be related to eolian sands identified in the Chad basin between 5.89 ± 0.19 Ma and 6.01 ± 0.23 Ma^35^. Other mechanisms for the variations in susceptibility are also possible and they could be related to variations in redox conditions or detrital influx^36^. A more detailed rock magnetic study would be necessary to solve this question. The FFT results can be best interpreted to reflect eccentricity modulation of precession together with a weak obliquity signal (Fig. 2). This results in a rough estimate of preserved time within the outcropping Vrysses Group at Trachilos Beach of ~ 200 kyr and an estimate of the sediment accumulation rate of ~ 12 cm/kyr. Using magnetostratigraphic results of the underlying Potamida section, which terminates at around 7 Ma in a reversed chron^19^, and our planktonic foraminifera constraints of a sedimentation age younger than 6.42 Ma, respectively 6.35 Ma, we correlate the identified normal polarity interval of the Trachilos section with the Messinian normal polarity chron C3An.1n between 6.272 and 6.023 Ma^29^ as maximum constraints, where the Trachilos section can also represent a much shorter time period.

We cannot rule out that the 8 m stratigraphic gap between ~ 5 and 13 m contains the reversed polarity Chron C3An.1r (6.386–6.272 Ma^29^) and that the lower part of the section (0–5 m) was deposited during Chron C3An.2n. This would imply a slightly lower sediment accumulation rate of ~ 7 cm/ka, which raises some inconsistency because with such a rate the end of C3An.1n should be reached and the top of the Trachilos section should therefore be of reversed polarity. Even if there is a slight change of sediment accumulation rate present to account for the inconsistency, the footprints would be of a very similar age in such a scenario of ~ 6.06 Ma.

We can also reasonably exclude recent normal-polarity remagnetization of the sediments due to the low mean ChRM inclination (44.0°), about 11° shallower than the (present-day) 55° predicted by the geocentric axial dipole model at the sampling site. The observed two ~ 100 ka eccentricity cycles agree with our interpretation and would imply an age of ~ 6.05 Ma for the footprints yielding an increase of the footprint age of more than 300 ka^6^.

The fact that the bipedal *Orrorin tugenensis*^37^ has been identified mainly within the reversed chron C3r, but also within the normal polarity chron C3An.1n^38^, reveals a temporal overlap between *Orrorin* and the Trachilos footprints. Recently Macchiarelli et al.^39^ have suggested, on the basis of femoral morphology, that *Sahelanthropus tchadensis* may not have been habitually bipedal. This intriguing suggestion has yet to be verified by further work on the skeletal remains from Chad, but, if correct, it implies that *Orrorin* and the Trachilos footprints may together constitute the oldest evidence for bipedal hominids.

Irrespective of the affinity of the Trachilos footprints to the hominin lineage, we have in this paper refined the age of these tracks by placing them on a more secure geochronological basis. This will allow future descriptive and comparative analyses to be based on a robust temporal framework, facilitating the evaluation of the true significance of the Trachilos footprints and their relationship to the hominin lineage.

## Methods

### Magnetostratigraphy

We collected a total of 51 samples along the section. Samples were drilled in the field with an electric battery drill mounted with a diamond bit and oriented with a magnetic compass. From each sample we trimmed a standard ~ 11 cm^3^ core-specimen (2.54 cm of diameter) for paleomagnetic analyses. In order to retrieve the vector components of the natural remanent magnetization (NRM), a pilot set of 12 specimen was subjected to either stepwise alternating field (AF) demagnetization up to a maximum field of 100 mT, or stepwise thermal demagnetization up to a maximum temperature of 500 °C (steps are indicated in Figure S2). After the pilot phase, all remaining specimens (39) where demagnetized thermally. AF demagnetization was performed with a 2-G longcore system including an in-line AF demagnetizer, while thermal demagnetization with an ASC single chamber paleomagnetic furnace. The NRM has been measured after each demagnetization step with a 2G Enterprises SQUID magnetometer. Reliable characteristic remanent magnetization (ChRM) was obtained from 31 specimens (61%). We estimated the orientation of the paleomagnetic directions by applying the principal component analysis of Kirschvink^40^ on vector end-points selected visually from the demagnetization diagrams (Fig. S2)^41^. In order to identify the magnetic carriers responsible for the ChRM we performed susceptibility vs. temperature measurement on a set of 5 representative specimens throughout the section. To obtain cyclostratigraphic information^42^, low field magnetic susceptibility was obtained from all measured samples throughout the section. All paleomagnetic experiments were carried out at the paleomagnetic laboratory of the University of Tuebingen within a magnetically shielded room designed and built by Wolfgang Rösler.

### Cyclostratigraphy

A Fast-Fourier transform (FFT) method was applied to the susceptibility dataset of the Trachilos section (Fig. 2, S3, S4). The spectral power used was the complex conjugate of the Fourier coefficients, normalized to unit mean power^43^. The significance was estimated using a Monte Carlo noise estimation for 1000 randomly generated time series, including a 95% confidence level^43^ and additionally using a multi-taper approach^44^. The two most prominent peaks were further analysed with a bandpass filter using a Gaussian window (Fig. 2).

### Rock magnetism

We selected a representative set of 8 weighted specimens from the trimmed ends of the oriented core samples. After imparting an anhysteretic remanent magnetization (ARM) with an alternating field (AF) decaying from a peak value of 100 mT and direct current (DC) field of 50 µT, the specimens were subjected to stepwise AF demagnetization with peak field ranging from 2 to 100 mT (Fig. 2c). ARM demagnetization curves are normalized and plotted altogether to estimate the median destructive field (MDF) of the ARM, which is defined as the AF field at which half of the magnetization is lost. After this analysis, the same set of samples was subjected to stepwise backfield isothermal remanent magnetization (IRM) acquisition: specimens were first magnetized with a pulse field of 700 mT and then stepwise magnetized and measured for the IRM up to a field of 700 mT applied on the opposite direction. This allows to estimate the coercivity of remanence (Bcr), the field at which the IRM = 0. All curves, which are shown normalized and plotted on a logarithmic field scale in Fig. 2d, have been rescaled to positive half values and then “unmixed” for their coercivity components using the MAX UnMix web-based software^45^ (http://shinyapps.its.carleton.edu/max-unmix/). This software allows interpolating the IRM data by means of skewed generalized Gaussian (SGG) functions curves^46,47^. In order to monitor the thermal dependence of the magnetic mineralogy during heating, magnetic susceptibility during heating up to 700 °C was continuously monitored on a set of 5 representative specimens with an AGICO MFK-1 Kappabridge. ARM and IRM analyses were performed at the paleomagnetic laboratory of the University of Bremen (Germany) by using the automated measuring system^48^, placed in line with a 2G Enterprises cryogenic magnetometer. The susceptibility variations during heating have been measured in the paleomagnetic laboratory of the University of Tübingen (Germany).

The MDF of the ARM ranges between 19 and 37 mT, with an average value of 28 mT. This is similar to values observed in modern sediments containing magnetotactic bacteria (MTB), which range from 30 to 50 mT^49,50^. The lowest value could be explained by the contribution of detrital multi-domain magnetite particles, which possess lower MDF of ARM^51^. Coercivity of remanence (B_cr_) values derived by isothermal remanence magnetization (IRM) curves are well clustered between 38 and 51 mT, with an average of 45 mT, in good agreement with proposed MTB values^50^. The IRM curves show some complexity (e.g., inset in Fig. 2d), but they are dominated by one low-coercivity component that is fully saturated by 300 mT. The presence of more contributors to the total IRM can be related detrital magnetic grains. The susceptibility vs. temperature curves are not reversible, with much higher susceptibility values during cooling (inset in Fig. 2c), likely indicating the formation of magnetite during heating from a Fe-rich mineral precursor (e.g., clay minerals)^52^.

### Biostratigraphy

Samples for biostratigraphy were washed to collect the > 125 μm fractions, following standard sieve techniques. Planktonic foraminifera from the > 125 μm sieved fraction were analyzed to determine the presence and relative abundance of recorded species. Abundance and dominant coiling direction ratios (dextral and sinistral) of *Neogloboquadrina acostaensis* were also determined by counting (n = 200) all the individuals in four representative samples of the 125 μm fraction.

### Detrital zircon geochronology

Approximately 0.5 kg of fresh sample was crushed in a jaw crusher. After this, the material was sieved for the < 400 µm fraction. Heavy mineral separation from the latter was done using LST (lithium heteropolytungstate in water) prior to magnetic separation in the Frantz isomagnetic separator. Zircon grains for U–Pb dating were selected by hand-picking under a binocular microscope (ZEISS Stemi 2000-C). In order to get a representative selection of the overall zircon population, as many zircon grains as possible of all sizes, colors and morphologic types were randomly picked from the sample^53,54^. Subsequently, the zircon grains were mounted in resin blocks and polished to approximately half their thickness in order to expose their internal structure. Thus, CL-imaging was performed using SEM coupled to a HONOLD CL-detector operating with a spot size of 450 nm at 20 kV.

#### U–Pb age determination and Th–U measurement via LA-ICP-MS

In order to avoid mixed U–Pb ages resulting from different late-to postmagmatic or metamorphic influences, spots for isotope analyses were preferentially set on monophase growth patterns. U–Th–Pb isotopic analyses took place at the GeoPlasma Lab, Senckenberg Naturhistorische Sammlungen Dresden and were carried out via LA-ICP-MS (Laser Ablation with Inductively Coupled Plasma Mass Spectrometry) techniques. Therefore, Element 2 XR instrument coupled to an ASI RESOlution SE S155 193 nm Excimer Laser System was utilized (for data see electronic supplement). For ablation, the mounts were put into a Laurin Technic S155 ablation cell, which enables sequential sampling of heterogeneous grains (e.g., growth zones) during time-resolved data acquisition. Single measurement of one spot is composed of approximately 15 s background acquisition followed by 30 s data acquisition. With respect to grain structure and size, the chosen spot sizes were set at 25 µm. Correction of common-Pb, based on the interference- and background-corrected ^204^Pb signal and a model Pb composition^55^, was carried out if necessary. Judgement of necessity for correction depended on whether the corrected ^207^Pb/^206^Pb lay outside the internal errors of the measured ratios. A U–Pb analysis is concordant when it overlaps within uncertainty with the Concordia. So, it seems to be appropriate to exclude results with a low level of concordance (^206^Pb/^238^U age/^207^Pb/^206^Pb age × 100), but very large errors that overlap with the Concordia from interpretation. Thus, an interpretation with respect to the obtained ages was done for all grains within the concordance interval of 90–110% (^206^Pb/^238^U age/^207^Pb/^206^Pb age × 100) which is often used e.g.,^56^. Discordant analyses were generally interpreted with caution. Finally, raw data were corrected for background signal, common-Pb, laser induced elemental fractionation, instrumental mass discrimination, depth- and time-dependant elemental fractionation of Pb/Th and Pb/U by use of an Excel® spread sheet program developed by Richard Roper and Axel Gerdes (Frankfurt Isotope and Element Research Centre (FIERCE), Institute of Geosciences, Johann Wolfgang Goethe-University Frankfurt, Frankfurt am Main, Germany). The given uncertainties were propagated by quadratic addition of the external reproducibility obtained from the reference zircon GJ-1 (~ 0.6% and 0.5–1.0% for the ^207^Pb/^206^Pb and ^206^Pb/^238^U, respectively) during individual analytical sessions and the within-run precision of each analysis. Each sequence started with two measurements of the GJ-1, one measurement on the Plešovice and one measurement of the 91,500 reference zircon, followed by 20 analyses of unknown grains, and so on. The obtained calculated Concordia ages for the reference zircon analyses were at GJ-1 603 ± 2 Ma (GJ-1, n_total_ = 34), at 337 ± 2 Ma (Plešovice, n_total_ = 17) and at 1065 ± 7 Ma (91,500, n_total_ = 17), which are in line with the published ages e.g.,^57–60^. Concordia diagrams (2σ error ellipses) and concordia ages (95% confidence level) were produced by using Isoplot/Ex 4.13^61^. The program Age Display^62^ was employed to generate frequency as well as relative probability plots. For zircons with ages older than 1 Ga, ^207^Pb/^206^Pb ages were taken for interpretation, the ^206^Pb/^238^U ages for younger grains. Further details on analytical protocol and data processing are reported in^63,64^.

## Supplementary Information

Supplementary Information.
