# Supplementary material for: Age constraints for the Trachilos footprints from Crete
Source: Sci Rep. 2021 Oct 11;11:19427. doi: 10.1038/s41598-021-98618-0 (PMC8505496; doi:10.1038/s41598-021-98618-0)
Supplement: Supplementary file 1 — Supplementary Information. [file 41598_2021_98618_MOESM1_ESM.docx]

Supplementary Materials for

Age constraints for the Trachilos footprints from Crete

Uwe Kirscher^1,2*^, Haytham El Atfy^1,3^, Andreas Gärtner^4^, Philipp Munz^1^, Edoardo Dallanave^5^, Grzegorz Niedźwiedzki^6^, Athanassios Athanassiou^7^, Charalampos Fassoulas^8^, Ulf Linnemann^4^, Mandy Hofmann^4^, Matthew Bennett^9^, Per Erik Ahlberg^6^, Madelaine Böhme^1,2^

*^1^Department of Geosciences, Eberhard Karls University, Tübingen, 72076 Tübingen, Germany*

*^2^Senckenberg Centre for Human Evolution and Palaeoenvironment, Tübingen, Germany*

*^3^Department of Geology, Faculty of Science, Mansoura University, 35516 Mansoura, Egypt*

*^4^Senckenberg Naturhistorische Sammlungen Dresden, Museum für Mineralogie und Geologie, Sektion Geochronologie, Königsbrücker Landstraße 159, 01109 Dresden*

*^5^Faculty of Geosciences, University of Bremen, Klagenfurterstr. 2-4, 28359 Bremen, Germany*

*^6^Department of Organismal Biology, Uppsala University, Norbyvägen 18A, 752 36 Uppsala, Sweden*

*^7^Hellenic Ministry of Culture and Sports, Ephorate of Palaeoanthropology-Speleology, Ardittou 34B, GR-11636 Athens, Greece*

*^8^University of Crete, Natural History Museum, 71409 Iraklion, Greece*

*^9^Department of Life and Environmental Sciences, Bournemouth University, Bournmouth, UK*

*Email: [uwe](mailto:uwe).kirscher@uni-tuebingen.de

**This PDF file includes:**

Figures S1 to S7

Supplementary Text

**
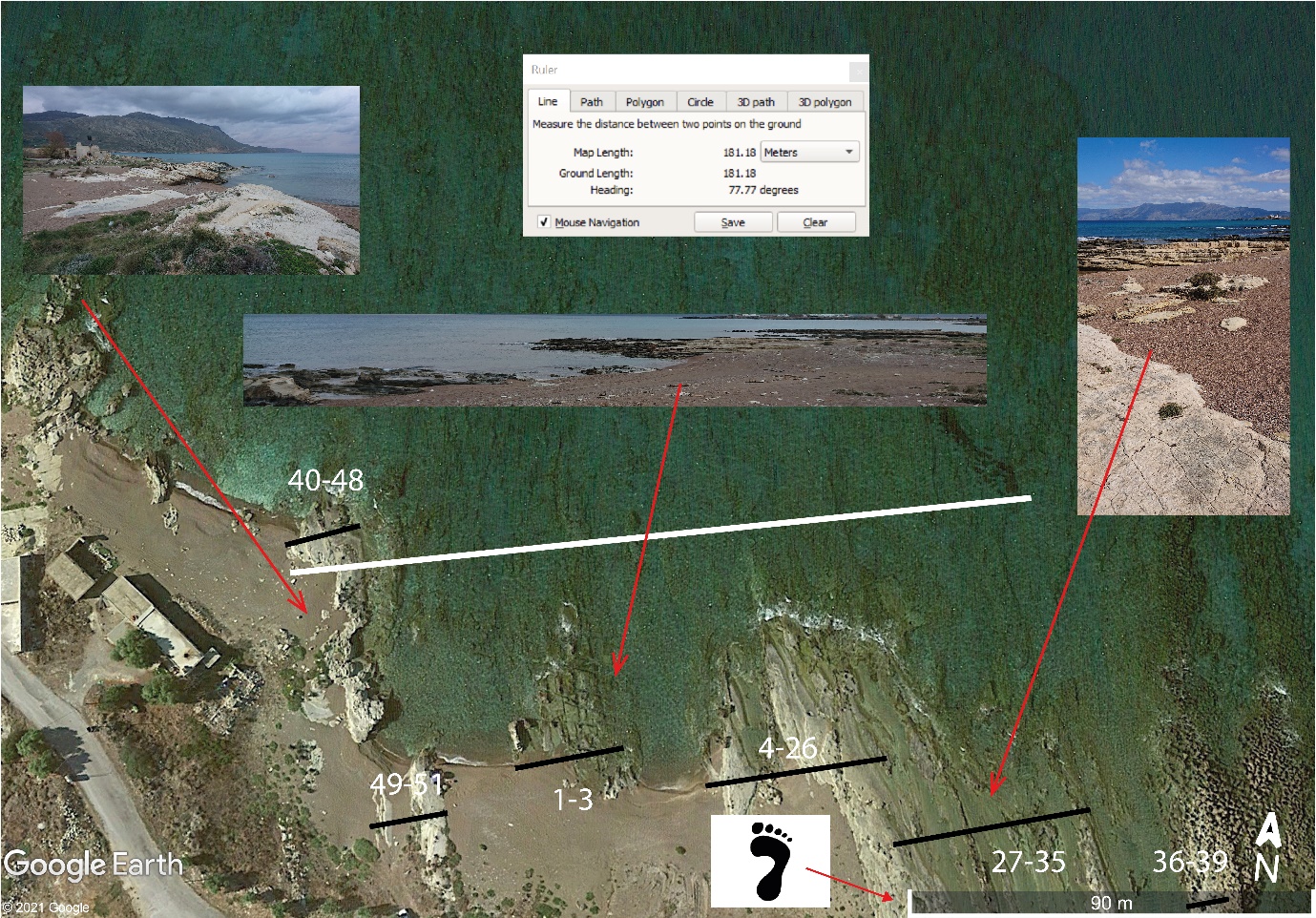
**

Figure S1: Google Earth image of the sampling site. White line represents section with ~13° dip. Pictures with red arrow show private field photographs. White numbers and black lines show places and numbers of paleomagnetic samples. Foot shows location of footprint site. This image is taken at Lat: ~35.515N° and Long: ~23.626°E.


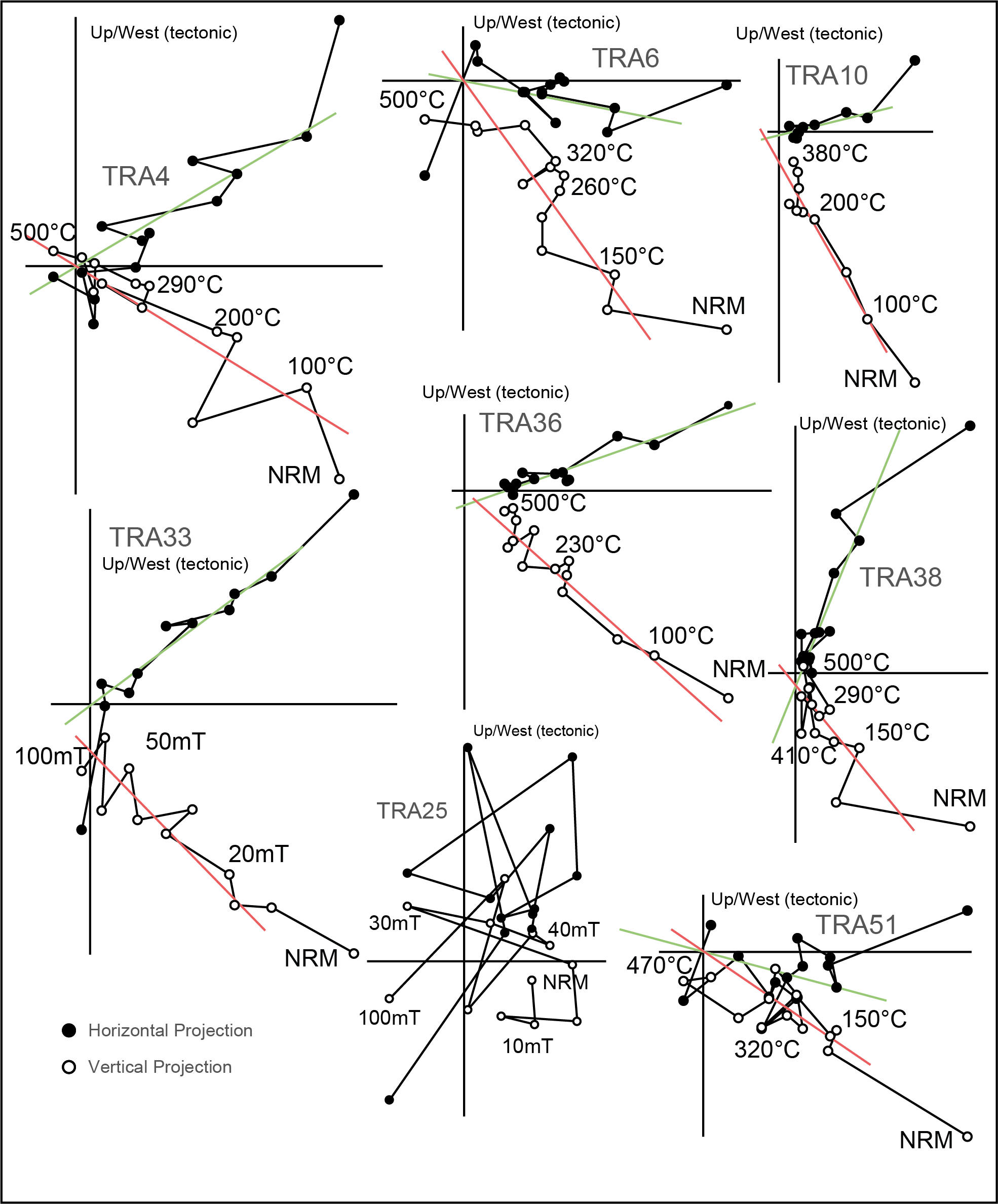


Figure S2: Representative demagnetization results shown on orthogonal vector endpoint diagrams^1^. Marked axis represents the up/down and west/east axis, unmarked axis reflects north/south projection. Open and closed circles indicate projections on the vertical and horizontal plane, respectively. Red and green lines show principal component analysis for both components. Figure and analysis were done using the online platform paleomagnetism.org^2^. TRA-25 is an example of a rejected sample.


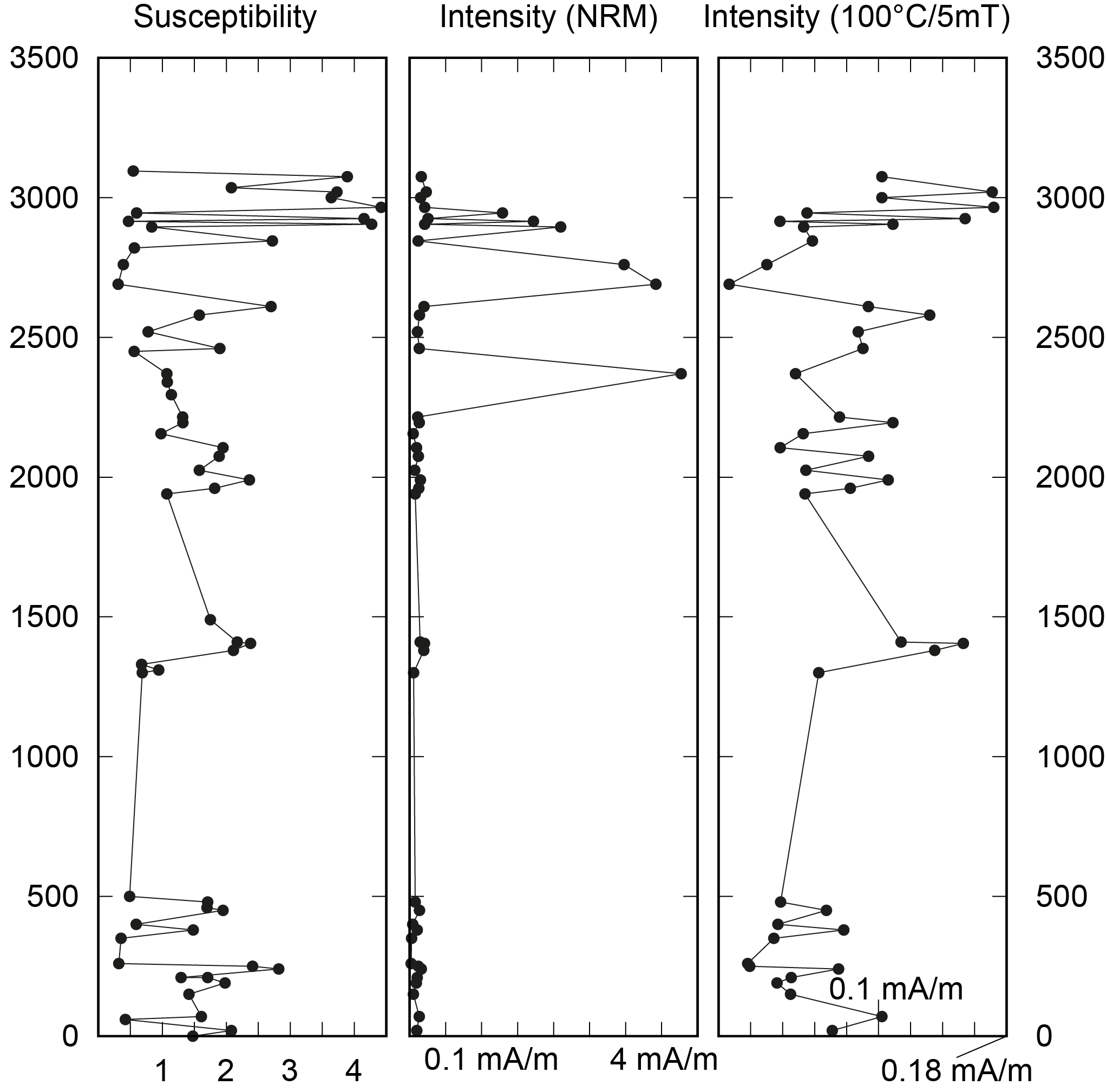


Figure S3: Susceptibility and magnetic intensity of the NRM and NRM demagnetized using 100°C thermal or 5 mT alternating field steps versus stratigraphic height in cm.

**
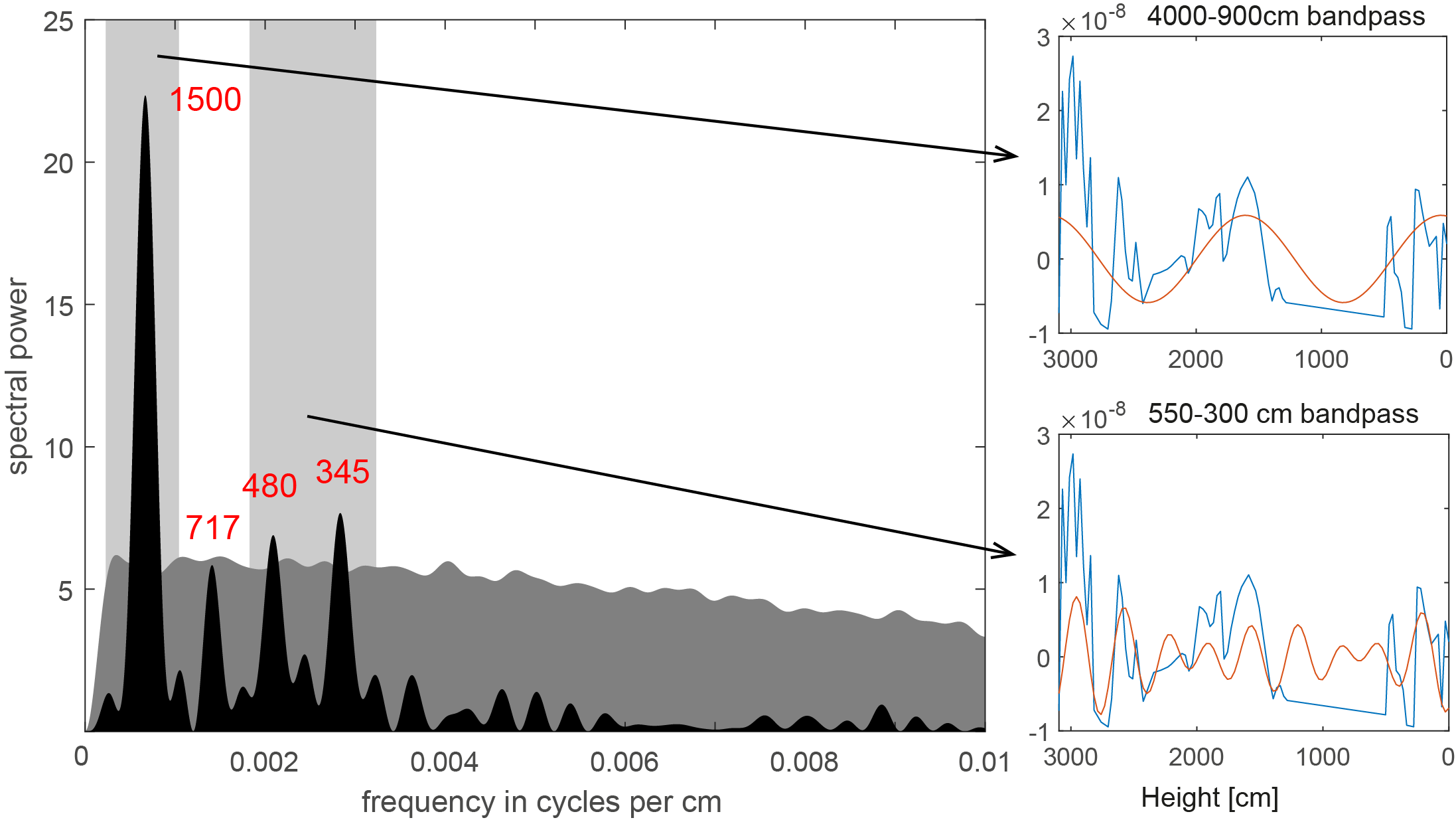
**

Figure S4: Spectrum and band-pass filters of spectral analysis of the magnetic susceptibility signal using the approach of ^3,4^. Left figure shows the spectrum with a 95% Monte Carlo noise estimate and right figures show Gaussian band pass filters of indicated bands.


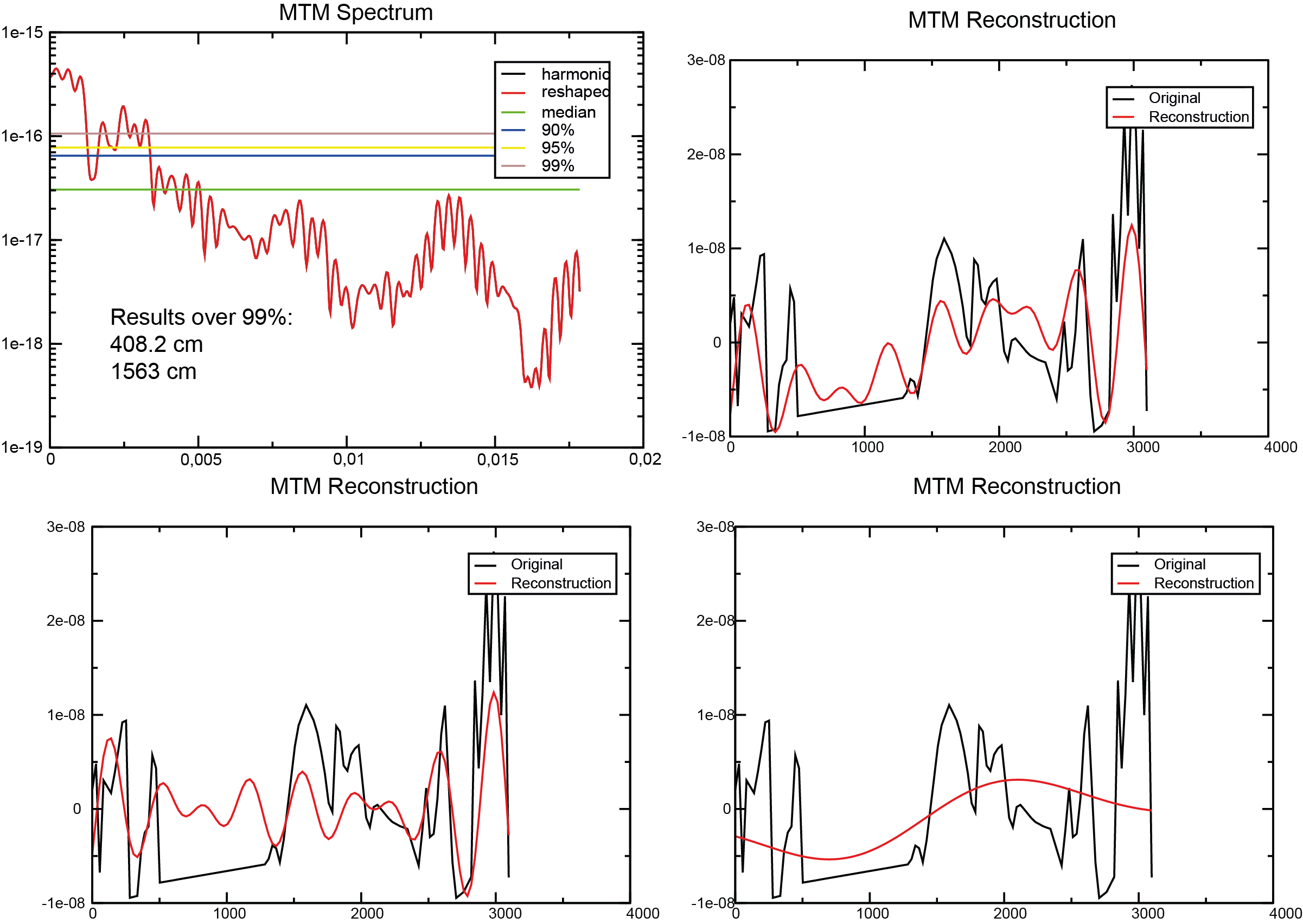


Figure S5: Additional spectral analysis using multi-taper method of Ghil, et al. ^5^ showing the spectra (top left), and reconstructions based on the two most prominent cycles above 99% confidence (top right shows the combination and lower figures show the separated two strongest peaks of 408.2 and 1563 cm).


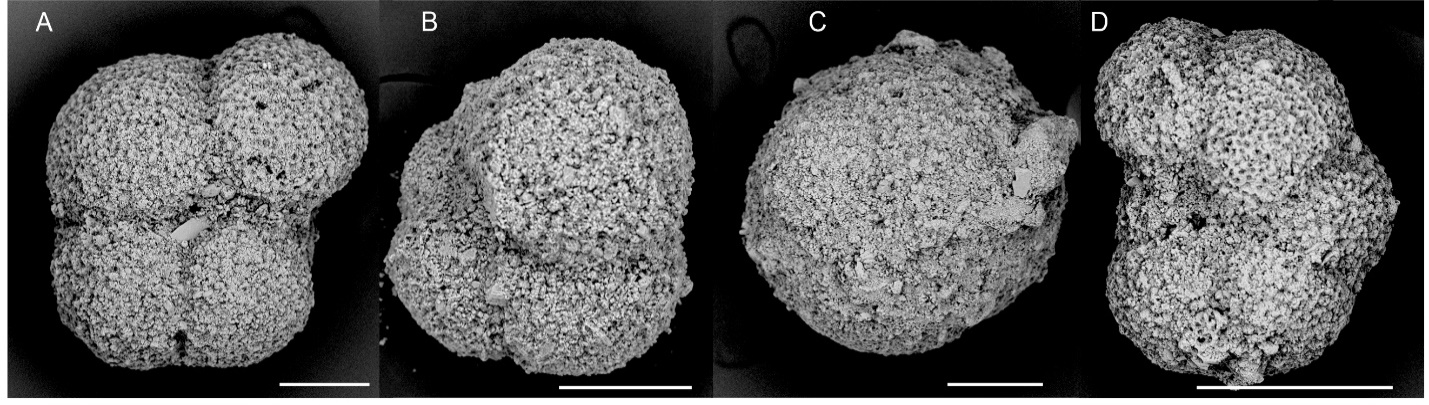


Figure S6: SEM examples of identified foraminifera. Bar scale is equal to 100 μm. A: *Neogloboquadrina acostaensis* sinistral, B: *Neogloboquadrina acostaensis* dextral, C: *Orbulina* sp., D: *Turborotalita multilobe.*

Figure S7: Detrital zircon geochronology results of Trachilos samples A, B, and C; binned frequency probability-density plots, bin width = 25 Ma. Colored curves represent concordant analyses, while discordant results are indicated by grey color.

# **SUPPLEMENTARY TEXT**

**GEOLOGY**

The Platanos Basin is the westernmost Neogene basin in NW-Crete. This tectonically active small basin is bounded by steep normal faults to the north, east and south and is open to the west. The Neogene succession is characterized by two sedimentary transgressive-regressive cycles of late Tortonian-early Messinian (Miocene) and early Piacenzian age (Pliocene), respectively ^6^. The sedimentary rocks deposited during the Miocene cycle measure up to 280 m ^7^. A great variety of continental to shallow marine sedimentary facies have been described ^8,9^, and the distribution depends on the position on swells or basins. Marginal and littoral sediments dominate the entire Platanos Basin and are up to 80-100 m thick at the Platanos section ^6,7^ in the southern part of the basin. This shallow marine facies is composed of alternating yellow marls, grey calcarenites and bioclastic (*Lithothamnium*, coralline algae) limestones and is named facies-type B or transitional facies by Frydas and Keupp ^6^ and Keupp and Bellas ^10^, and heterolithic facies by Kontopoulos, et al. ^7^. With this facies the Miocene cycle in the Platanos Basin terminates ^6^.

The upper 50 meters of the heterolithic facies in the main Plantanos section 7 km to the South of the Trachilos Beach contain calcareous nannoplankton of biozone CN9bB (total range of *Nicklithus amplificus* sensu Raffi, et al. ^11^, top C3Ar to C3An/C3r, 6.727-6.023 Ma) with common occurrence of *N. amplificus* together with few *Amaurolithus delicatus* at its base ^6^. *A. delicatus* has its first occurrence in the Mediterranean Basin at 6.415 Ma ^12^, restricting the age of the upper 50 meters of Miocene sediments in the Platanos Basin to 6.4-6.0 Ma. During the CN9bB (*N. amplificus* total range biozone), a coiling change of the planktonic foraminifer *Neogloboquadrina acostaensis* from sinistral to predominantly dextral is recognized in the Mediterranean Basin at 6.35 Ma ^13^.

Miocene sediments at Trachilos Beach dip with 8 to 12 degrees towards ENE. Over a distance of 250 meters along the beach line, between E 23°37‘and 32‘‘ to 40‘‘, a 30.5 m thick profile is exposed. Further below the profile is covered by modern beach sand and above by cemented Holocene beach conglomerates.

The sedimentary rocks represent a fairly regular decimetre-scale alternation of 1) yellow silty marls, containing few centimeters thick layers of beige calcarenites, 2) grey intraclastic calcarenites with erosive bases, abundant fragments of coralline algae and chaotic orientation of clasts from yellow marls and calcarenites, and 3) bioclastic and partially laminated calcarenites with rare coralline algae and a low density of trace fossils (e.g. *Thalassinoides* and meniscate echinoid burrows). This lithofacies development is, with the exception of the up to one meter thick grey intraclastic calcarenites, interpreted as Tsunamites by Gierliński, et al. ^14^, very similar to the heterolithic facies described by Kontopoulos, et al. ^7^ from the top 80 meters of the Platanos section 7 km to the south.

REFERENCES

1 Zijderveld, J. D. A. A. C. demagnetization of rocks: Analysis of results. *in: Collinson, D.W., Creer, K.M., Runcorn, S.K. (Eds.), Methods in Paleomagnetism. Elsevier, Amsterdam*, 254-286 (1967).

2 Koymans, M. R., van Hinsbergen, D. J. J., Pastor-Galán, D., Vaes, B. & Langereis, C. G. Towards FAIR Paleomagnetic Data Management Through Paleomagnetism.org 2.0. *Geochemistry, Geophysics, Geosystems* **21**, e2019GC008838, doi:10.1029/2019gc008838 (2020).

3 Muller, R. A. & MacDonald, G. J. *Ice ages and astronomical causes: data, spectral analysis and mechanisms*. (Springer Science & Business Media, 2002).

4 Mitchell, R. N. *et al.* Harmonic hierarchy of mantle and lithospheric convective cycles: Time series analysis of hafnium isotopes of zircon. *Gondwana Res* **75**, 239-248, doi:10.1016/j.gr.2019.06.003 (2019).

5 Ghil, M. *et al.* Advanced spectral methods for climatic time series. *Rev Geophys* **40**, 1003, doi:10.1029/2000rg000092 (2002).

6 Frydas, D. & Keupp, H. Biostratigraphical results in Late Neogene deposits of NW Crete, Greece, based on calcareous nannofossils. *Berliner Geowissenschaftliche Abhandlungen*, 169-189 (1996).

7 Kontopoulos, N., Zelilidis, A. & Frydas, D. Late Neogene sedimentary and tectonostratigraphic evolution of northwestern Crete island, Greece. *Neues Jahrbuch für Geologie und Paläontologie-Abhandlungen*, 287-311 (1996).

8 Freudenthal, T. *Stratigraphy of Neogene deposits in the Khania Province, Crete, with special reference to foraminifera of the family Planorbulinidae and the genus Heterostegina*, Utrecht University, (1969).

9 Meulenkamp, J. E. *Field Guide to the Neogene of Crete*. (Department of Geology and Paleontology, University of Athens, 1979).

10 Keupp, H. & Bellas, S. Neogene development of the sedimentary basins of NW Crete island, Chania Prefecture, South Aegean Arc System (Greece). *Berliner Geowiss. Abh.* **34**, 3-117 (2000).

11 Raffi, I. *et al.* A review of calcareous nannofossil astrobiochronology encompassing the past 25 million years. *Quaternary Sci Rev* **25**, 3113-3137 (2006).

12 Manzi, V. *et al.* Age refinement of the Messinian salinity crisis onset in the Mediterranean. *Terra Nova* **25**, 315-322 (2013).

13 Lirer, F. *et al.* Mediterranean Neogene planktonic foraminifer biozonation and biochronology. *Earth-Science Reviews* **196**, 102869 (2019).

14 Gierliński, G. D. *et al.* Possible hominin footprints from the late Miocene (c. 5.7 Ma) of Crete? *P Geologist Assoc* **128**, 697-710, doi:10.1016/j.pgeola.2017.07.006 (2017).
